# Biosimilar Uptake in Medicare Advantage vs Traditional Medicare

**DOI:** 10.1001/jamahealthforum.2023.4335

**Published:** 2023-12-28

**Authors:** Steven Kozlowski, Andrew Kwist, Rowan McEvoy, Nirabh Koirala, Yoganand Chillarige, Jeffrey A. Kelman, David J. Graham

**Affiliations:** 1 Center for Drug Evaluation and Research, US Food and Drug Administration, Silver Spring, Maryland; 2 Acumen LLC, Burlingame, California; 3 Centers for Medicare & Medicaid Services, Washington, DC

## Abstract

This cross-sectional study uses Traditional Medicare and Medicare Advantage claims data to evaluate uptake of biosimilars relative to their reference products.

## Introduction

Biosimilar products can increase access and reduce treatment costs for chronic and life-threatening diseases. As of March 1, 2023, 40 biosimilar products were approved by the US Food and Drug Administration (FDA).^1^ Biosimilar uptake has varied, potentially due to factors such as health care setting, pricing, and market entrance timing.^2^

Medicare Advantage tools including preauthorization and payment practices encourage insurers’ use of lower-cost alternatives.^3^ To assess differences in biosimilar use between Medicare Advantage (MA, managed care Part C) and traditional Medicare (TM, fee-for-service Part B), we examined uptake of biosimilars relative to their reference products.

## Methods

Using all administrative TM claims and MA encounter data from May 2015 through September 2022, we evaluated biosimilar market share for products with at least 1 biosimilar administration on or before September 30, 2019, for this cross-sectional study. We included 20 total biosimilar products across 7 product types with use mostly in Parts B and C. Within each product type, market share was calculated cumulatively through 36 months after biosimilar introduction, defined as the number of biosimilar administrations of total administrations of biosimilars and their reference products. We further stratified biosimilar market share by indication for bevacizumab and epoetin alfa. This study was approved as a surveillance activity by the FDA/Center for Drug Evaluation and Research institutional review board liaison and followed STROBE reporting guidelines. Analyses were performed using R version 4.1.2.

## Results

**Figure 1.**
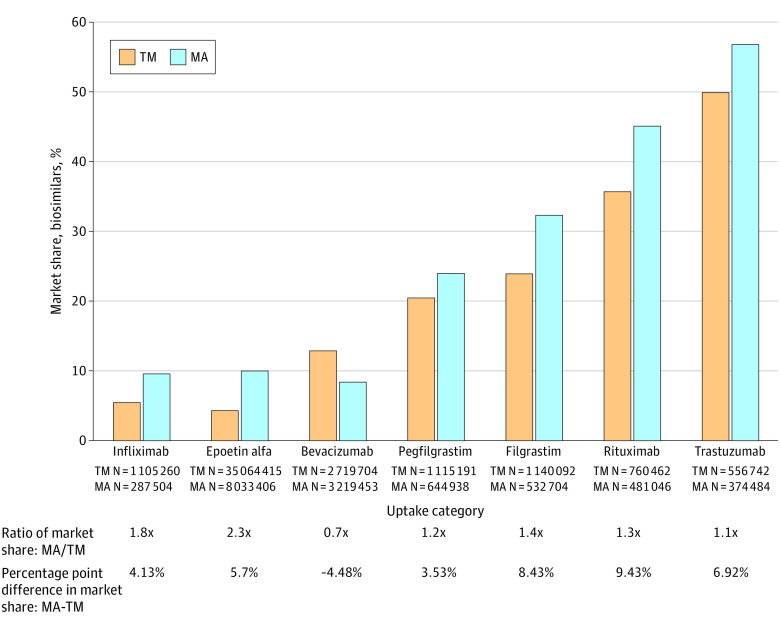
Cumulative Percentage Market Share of Biosimilars Through 36 Months After Biosimilar Introduction, Stratified by Traditional Medicare (TM) vs Medicare Advantage (MA) The percentage of the market share held by all aggregated biosimilar products of all usage of that product type (all marketed biosimilars, and their reference products, including tbo-filgrastim, approved under the US Food and Drug Administration full Biologics License Application before establishment of the biosimilar approval pathway^6^) through 36 months after biosimilar introduction, stratified by TM vs MA. Product administrations were identified via Healthcare Common Procedure Coding System codes and modifiers in Part B claims and Part C encounter data.

Biosimilar uptake was greater in MA than TM for 6 of 7 product types, ranging from 1.1 times greater for trastuzumab to 2.3 times greater for epoetin alfa (Figure 1). The median MA to TM ratio across all product types was 1.3 (range, 0.7-2.3), and median difference was 5.7% (range, −4.48% to 9.43%). We further investigated 2 product types: (1) bevacizumab, whose biosimilar market share was lower in MA than TM, and (2) epoetin alfa, whose biosimilar market share in MA was more than 2 times greater than TM.

**Figure 2.**
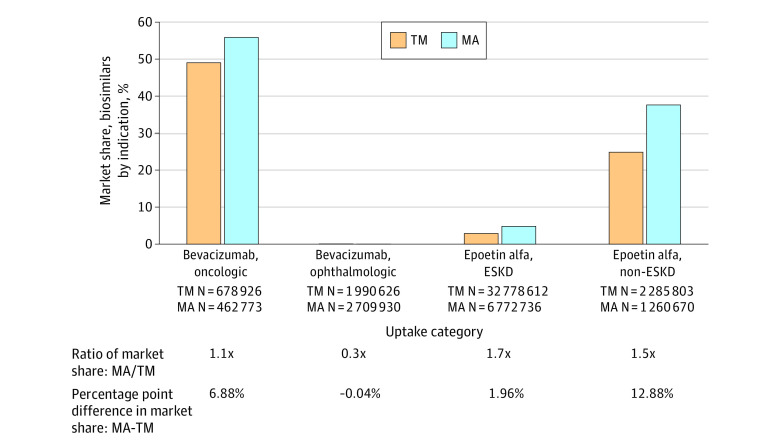
Cumulative Percentage Market Share of Bevacizumab and Epoetin Alfa Biosimilars Through 36 Months After Biosimilar Introduction, Stratified by Indication and Traditional Medicare (TM) vs Medicare Advantage (MA) The percentage of the market share held by all aggregated biosimilar products out of all bevacizumab and epoetin alfa product usage through the first 36 months after biosimilar introduction, stratified by TM vs MA. Bevacizumab usage is stratified by ophthalmic and oncologic indications, using ophthalmic-related and oncologic-related keywords in the administrations’ corresponding primary diagnoses codes to categorize administrations (approximately 2% of total administrations did not have either type of keyword and were excluded). Epoetin alfa usage is stratified by end-stage kidney disease (ESKD) with dialysis vs non-ESKD, using administrations’ Healthcare Common Procedure Coding System code description to categorize administrations.

Given the off-label bevacizumab indication for age-related macular degeneration,^4^ we stratified by ophthalmic vs oncologic usage (Figure 2). For ophthalmic, biosimilar use was very low but market share was higher in TM. For oncologic, biosimilar use was 1.1 times greater in MA than TM, aligned with other product types. This suggests the apparent greater biosimilar use in TM when aggregated across indications is due to the differential dilution of market share by the large overall proportion of ophthalmic usage.

For epoetin alfa, as product decisions vary for patients with end-stage kidney disease (ESKD), we stratified usage by ESKD vs non-ESKD (Figure 2). Within each indication, biosimilar market share was 1.5 to 1.7 times greater in MA than TM, similar to most other product types. The large 2.3 times difference when aggregated across indications is due to higher relative weighting of MA biosimilar use when combining ESKD and non-ESKD.

## Discussion

After accounting for some differences due to indication, biosimilar market share was consistently greater in MA than TM, aligned with other studies supporting more lower cost prescribing substitution in MA.^3^

A study of lower cost uptake in Medicaid of an insulin biosimilar and complex generic found uptake was more than 10 times higher in managed care vs fee-for-service Medicaid.^5^ One suggested explanation was differential statutory state inflation rebates in managed care vs fee-for-service payers. Implementing the Medicare inflationary rebate may also affect MA and TM biosimilar uptake.

The higher MA biosimilar uptake observed in our study may be affected by differences in population characteristics between TM and MA.^3^ Further studies are needed that account for these covariates and more specific clinical indications.

Current estimates of biosimilar savings are greater than $20 billion,^6^ meaning each percent increase in overall biosimilar uptake could represent hundreds of millions of dollars. Thus, it is important to validate the increased biosimilar uptake observed in MA and investigate potential mechanisms through which managed care may encourage greater biosimilar use.

## Notes

Supplement.
Data Sharing Statement
