# Supplementary material for: Biosimilar Uptake in Medicare Advantage vs Traditional Medicare
Source: JAMA Health Forum. 2023 Dec 28;4(12):e234335. doi: 10.1001/jamahealthforum.2023.4335 (PMC10755621; doi:10.1001/jamahealthforum.2023.4335)
Supplement: Supplement. — Data Sharing Statement [file jamahealthforum-e234335-s001.pdf]

Data Sharing Statement

Kozlowski. Biosimilar Uptake in Medicare Advantage vs Traditional Medicare. *JAMA Health Forum*. Published December 28, 2023. doi:10.1001/jamahealthforum.2023.4335

Data
Data available: No

Additional Information
Explanation for why data not available: Individual patient data is protected information. The authors can be contacted for access to summary data.
